# Paroxysmal Tachycardia Diagnosed by ECG247 Smart Heart Sensor in a Previously Healthy Child

**DOI:** 10.1155/2022/9027255

**Published:** 2022-03-27

**Authors:** Jarle Jortveit, Andreas Früh, Hans Henrik Odland

**Affiliations:** ^1^Sorlandet Hospital, Department of Cardiology, Arendal, Norway; ^2^Department of Paediatric Cardiology, Oslo University Hospital, Oslo, Norway

## Abstract

Supraventricular tachycardia (SVT) is the most common symptomatic heart rhythm disorder in children and adolescents. ECG recordings of the heart rhythm during episodes is necessary for the diagnosis and for the selection of treatment. However, conventional long-term ECG recording systems may miss the diagnosis due to the disease's intermittent nature. Novel adhesive patch ECG monitors, like ECG247 Smart Heart Sensor, may represent new important diagnostic tools in children and adolescents with symptoms of heart rhythm disorders. We report a case of tachyarrhythmia in a previously healthy 12-year-old child.

## 1. Introduction

Tachycardia is common in children, and the aetiology is usually benign. However, episodes of tachycardias may be caused by cardiac arrhythmias, and complaints of tachycardia require assessment of the cardiac rhythm. The most common symptomatic heart rhythm disorder in children and adolescents is supraventricular tachycardia (SVT), but any type of arrhythmias including life-threatening diseases may occur. An ECG recording during symptoms is crucial for correct diagnosis and for guidance of further assessment and treatment. However, ECG recording during an episode of arrhythmia can be difficult to obtain due to the intermittent nature of the condition. Equipment for long-term ECG recording, often referred to as “Holter monitoring,” has been in clinical use since the 1960s [1]. Prolonged ECG recording may increase the diagnostic yield of ECG tests [2]. Available equipment for long-term ECG diagnostics is developed and adapted for adults, and many patients find the ambulatory Holter monitor uncomfortable to wear during daily activities [3, 4]. Novel adhesive patch ECG monitors, like ECG247 Smart Heart Sensor, may represent new important diagnostic tools in children and adolescents with symptoms of heart rhythm disorders [5, 6].

Here, we report a previously healthy child with intermittent episodes of tachycardia.

## 2. Case Presentation

A twelve-year-old boy presented to the cardiology out-patient clinic due to episodes of tachycardia and palpitations. He had shown normal growth and development and was previously healthy. He played football and was physically very active. His father and grandfather had atrial fibrillation, but there were no other known heart diseases in the family.

The symptoms had lasted for several years, but the frequency of the episodes had increased from 1 to 2 episodes per year to 0–4 episodes per month. He described palpitations, headache, nausea, and chest pain during the episodes of tachycardia. He became tired and could not bear to participate in physical activity. The symptoms lasted from a few minutes to up to one hour. The parents had tried to count the heart rate during seizures and estimated the heart rate at about 180 beats per minute. He had seen his GP several times for symptoms, but the symptoms had disappeared before an ECG recording was performed.

**Figure 1:**
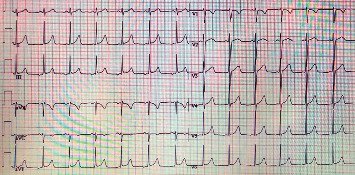
12-Lead ECG from the patient.

The patient's height and weight were normal in relation to age. He had no murmur over the heart. ECG showed an ectopic atrial rhythm, 72 beats/min, and was otherwise considered normal (Figure 1). No ventricular preexcitation pattern on ECG was identified.

Echocardiographic examination showed a structurally normal heart with normal dimensions and normal systolic ventricular function. There was no valve pathology and no shunts. Blood tests for the thyroid function were normal.

**Figure 2:**
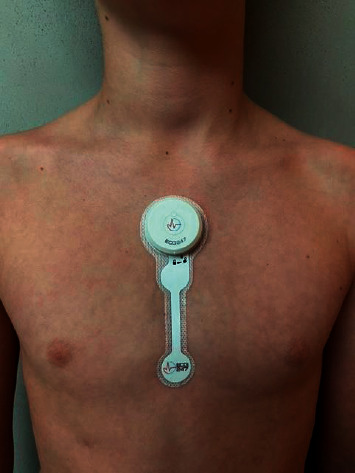
The ECG247 Smart Heart Sensor attached to the patient's chest.

The patient was demonstrated the use of ECG247 Smart Heart Sensor (Figure 2) and brought a sensor home for use in case of new episodes of tachycardia.

**Figure 3:**
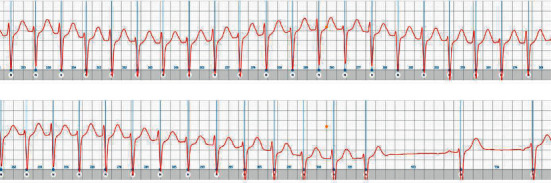
ECG recording from the patient during an episode of tachycardia (50 mm/s).

A few days later, he reported a new tachycardia attack while in school. The 12-year-old child immediately adhered the sensor over sternum and started the ECG recording via his mobile phone. His parents then notified the cardiologist at the hospital who assessed the patient's ECG recordings shortly afterwards via a remote web application. The ECG showed a narrow complex regular rhythm with RR intervals of 260–280 ms with transition to normal sinus rhythm after about 20 minutes (Figure 3).

Electrophysiological examination confirmed an atrioventricular reentrant tachycardia (AVRT) which was successfully catheter ablated.

## 3. Discussion

Supraventricular tachycardia (SVT) is defined as an abnormal rapid heart rhythm originating above the ventricles [7]. SVT is usually characterized with narrow QRS complexes on ECG. SVT is the most frequent heart rhythm disorder in children and adolescents, with an estimated prevalence of 0.1–0.4% in the paediatric population [8]. Atrioventricular reentrant tachycardia (AVRT) is the most common form of SVT followed by atrioventricular nodal reentrant tachycardia (AVNRT) [9]. The majority of children and adolescents with SVT have normally structured hearts, but the risk of SVT is increased in children and adolescents with congenital heart diseases [10]. A reentrant rhythm involves two distinct pathways for conduction between the atria and the ventricles, which creates a circuit through which an electrical impulse can cycle repetitively in one direction. Symptoms of SVT in children and adolescents include palpitations, chest discomfort, fatigue, and lightheadedness [11]. SVT is characterized by abrupt onset and termination. Most SVT episodes occur at rest. Some episodes last only one to two minutes, while others persist for hours, and the intervals between episodes show great variation from several years to daily [8]. Typical heart rate during SVT ranges from 180 to 240 beats per minutes in children and adolescents. Most children and adolescents tolerate episodes of SVT well. Sudden cardiac death is rare in patients with SVT, but the risk is higher in patients with congenital heart disease and in patients with ventricular preexcitation (Wolff–Parkinson–White (WPW) pattern on ECG, short PR-interval and delta wave) [11, 12].

Since most children and adolescents with SVT have sporadic brief episodes of arrhythmia, it may be difficult to capture an episode on ECG. Holter monitors allow continuous ECG recording for typical 24–48 hours and is therefore useful only in patients with frequent runs of SVT. Adhesive patch monitors allowing for longer heart rhythm monitoring may be an excellent alternative to Holter monitors, especially in children and adolescents [13, 14]. Sensors intended for self-testing are especially useful in cases with short and/or rare episodes. Implantable loop recorders (ILR) allow for continuous rhythm monitoring over years, but such devices require invasive procedures with high cost and potential risk. ECG documentation is necessary for the documentation of arrhythmias, and “smart watches” with heart rate monitoring based on arterial pressure waves are not sufficient.

The ECG247 Smart Heart Sensor (AppSens, Lillesand, Norway), originating from Sorlandet Hospital, Arendal, Norway, and University of Agder, Grimstad, Norway, is a wireless single-lead patch ECG monitoring device system consisting of an electrode patch with a single-use battery, a reusable sensor, a smartphone application, a back-end cloud service, and a web portal. The water-resistant sensor attaches over the sternum and continuously monitors the heart rhythm for up to 14 days without need for charging. All ECG recordings are sent from the ECG247 sensor through a dedicated application on the patient's mobile phone to a secure cloud storage solution with remote real-time web-access for healthcare professionals. The sensor has a flash memory for temporary storage of ECG data in case of loss of Bluetooth communication with the patient's smartphone, but a smartphone with Internet access is required to conduct the test. A patented dedicated shielding system is incorporated into the ECG247 electrode patch to protect from electrostatic discharges, and high-quality ECG signals can be obtained even if a shirt rubbing against the sensor surface during daily activities [5]. The ECG247 Smart Heart Sensor has incorporated algorithms based on artificial intelligence (AI) for detections of arrhythmias with high diagnostic accuracy [6]. The ECG247 Smart Heart Sensor is easy to use and has significantly improved usability compared to conventional Holter systems [6]. The system follows the requirements given by the General Data Protection Regulation (GDPR) and is certified as a medical diagnostic device class II, according to the EU Medical Device Directives (93/42/EEC).

A single-lead ECG may be more difficult to interpret compared to a multilead ECG. However, the number of leads is less important for the interpretation of narrow QRS complex arrhythmias like SVT. Arrhythmias characterized by a shift in the electrical axis and/or altered QRS morphology, e.g., ventricular tachycardia, are very rare in children and adolescents.

In conclusion, ECG documentation of the heart rhythm during symptoms is necessary for the diagnosis and for the guiding of therapy in patients with episodes of tachycardia and/or palpitations. The novel ECG247 Smart Heart Sensor allows for long-term ECG monitoring during activity with remote real-time access to the ECG recordings and may represent a new opportunity to improved diagnostics in children and adolescents with symptoms of heart rhythm disturbances.

## Data Availability

The data used to support the findings of this study are included within the article.
